# Identification of *Mycobacterium *spp. of veterinary importance using *rpoB *gene sequencing

**DOI:** 10.1186/1746-6148-7-77

**Published:** 2011-11-25

**Authors:** James Higgins, Patrick Camp, David Farrell, Doris Bravo, Mateja Pate, Suelee Robbe-Austerman

**Affiliations:** 1 Mycobacteria and Brucella Section, National Veterinary Services Laboratories, USDA-APHIS, 1920 Dayton Ave, Ames, IA 50010, USA; 2 Veterinary Faculty, Institute of Microbiology and Parasitology, University of Ljubljana, Ljubljana, Slovenia

## Abstract

**Background:**

Studies conducted on *Mycobacterium *spp. isolated from human patients indicate that sequencing of a 711 bp portion of the *rpoB *gene can be useful in assigning a species identity, particularly for members of the *Mycobacterium avium *complex (MAC). Given that MAC are important pathogens in livestock, companion animals, and zoo/exotic animals, we were interested in evaluating the use of *rpoB *sequencing for identification of *Mycobacterium *isolates of veterinary origin.

**Results:**

A total of 386 isolates, collected over 2008 - June 2011 from 378 animals (amphibians, reptiles, birds, and mammals) underwent PCR and sequencing of a ~ 711 bp portion of the *rpoB *gene; 310 isolates (80%) were identified to the species level based on similarity at ≥ 98% with a reference sequence. The remaining 76 isolates (20%) displayed < 98% similarity with reference sequences and were assigned to a clade based on their location in a neighbor-joining tree containing reference sequences. For a subset of 236 isolates that received both 16S rRNA and *rpoB *sequencing, 167 (70%) displayed a similar species/clade assignation for both sequencing methods. For the remaining 69 isolates, species/clade identities were different with each sequencing method. *Mycobacterium avium *subsp. *hominissuis *was the species most frequently isolated from specimens from pigs, cervids, companion animals, cattle, and exotic/zoo animals.

**Conclusions:**

*rpoB *sequencing proved useful in identifying *Mycobacterium *isolates of veterinary origin to clade, species, or subspecies levels, particularly for assemblages (such as the MAC) where 16S rRNA sequencing alone is not adequate to demarcate these taxa. *rpoB *sequencing can represent a cost-effective identification tool suitable for routine use in the veterinary diagnostic laboratory.

## Background

Skin and tissue infections caused by *Mycobacterium *spp. constitute a significant animal health problem for veterinarians involved in the care of livestock and companion animals. Of particular concern are the species in the *Mycobacterium avium *complex (MAC). While the taxonomy of the MAC has undergone some revision over the past decade, currently it is considered to comprise *M. avium *subsp. *avium*, *M. avium *subsp. *paratuberculosis, M. avium *subsp. *hominissuis*, *M. avium *subsp. *silvaticum*, *M. intracellulare*, *M. chimaera*, *M. colombiense*, *M. arosiense*, and *M. vulneris *[1-5]. Recently Ben Salah et al [6] designated three new species isolated from clinical cases in southern France, *Mycobacterium marseillense*, *Mycobacterium timonense*, and *Mycobacterium bouchedurhonense*, as members of the MAC.

While 16S rRNA sequencing has some utility for identifying species of *Mycobacterium*, some subspecies/species within the MAC share identical 16S rRNA sequences and thus cannot be differentiated using this locus [7]. Accordingly, a variety of alternate targets have been investigated for use in differentiating MAC species. One of the better characterized targets for bacteria, including *Mycobacterium*, is the RNA polymerase β- subunit (*rpoB*) gene [8]. Initial efforts in using the *rpoB *gene as a target for PCR and sequencing-based differentiation among *Mycobacterium *spp. were reported by Kim et al. [9,10]. Further studies describing the use of this locus by Adekambi et al. [11] and Adekambi and Drancourt [12] established the presence of multiple nucleotide polymorphisms in the full-length *rpoB *gene sequence among mycobacteria.

Ben Salah et al. [13] identified a 711-bp region of the *rpoB *gene harboring the greatest number of such polymorphisms, and used sequences from this region to investigate a panel of 100 clinical isolates provisionally assigned as MAC. These authors reported successfully assigning 93 of the isolates to a species/subspecies classification. Simmon et al. [14] used simultaneous sequencing of *rpoB *and 16S rRNA loci to identify clinical isolates of *Mycobacterium*; of 139 isolates, 117 (84%) were identified to the species level. More recently, Whang et al. [15] used a combination of *rpoB *PCR, followed by restriction fragment length polymorphism, to identify members of the MAC among 185 isolates, including 68 samples of ruminant origin.

The majority of published studies on the use of *rpoB *sequencing to identify mycobacteria have been conducted on panels of clinical (i.e., human medicine) isolates, and limited collections of animal isolates. Accordingly, we were interested in evaluating the protocol of Ben Salah et al. [13] for the identification of a large number of isolates originating from a variety of animal species. We were particularly interested in the ability of the *rpoB *sequence to differentiate between *M. avium *subsp. *avium*, *M. avium *subsp. *paratuberculosis*, and *M. avium *subsp. *hominissuis*, as these are prominent pathogens of veterinary importance. Here, we report the results of our investigation into the use of *rpoB *sequencing to characterize 386 isolates generated from submissions to the National Veterinary Services Laboratories (NVSL) from companion animals, livestock, feral animals, and zoo animals.

## Results

### *rpoB *gene identification of veterinary isolates

A total of 386 isolates, collected over 2008 - June 2011 from 378 animals (amphibians, reptiles, birds, and mammals) underwent PCR and sequencing of a ~ 730 bp portion of the *rpoB *gene; 310 isolates (80%) were identified to the species level based on similarity at ≥ 98% with a reference sequence. The remaining 76 isolates (20%) displayed < 98% similarity with reference sequences and were assigned to a clade based on their location in a neighbor-joining tree containing reference sequences. The two most frequently encountered clades within the MAC included the clade containing *M. avium *subsp. *avium*, *M. avium *subsp. *paratuberculosis, M. avium *subsp. *silvaticum*, and *M. avium *subsp. *hominissuis*; and the clade containing *M. intracellulare*, *M. chimaera*, and *M. indicus pranii*.

For a subset of 236 isolates that received both 16S rRNA and *rpoB *sequencing, 167 (70%) displayed a similar species/clade assignation for both sequencing methods. For the remaining 69 isolates, species/clade identities were different with each sequencing method (this information is provided in Additional file 1).

### *rpoB *gene sequence of *M. avium *subsp. *silvaticum *ATCC 49884

We observed that two separate lots of the *M. avium *subsp. *silvaticum *type strain ATCC 49884, one lot purchased from that company prior to 2008, and the other in Spring 2011, yielded *rpoB *sequence with nucleotide 2, 541 (using the *M. avium *subspecies *paratuberculosis *rpoB K10 strain numbering convention) as a cytosine, rather than the thymine present in the existing GenBank deposition for *M. avium *subsp. *silvaticum *ATCC 49884 [GenBank: EF521905]. The electropherograms for this portion of the *rpoB *sequence displayed satisfactory peak height (Additional file 2), indicating that the cytosine call was not an artifact of sequencing chemistry. Subsequently, Ion Torrent - based genomic sequencing of the ATCC 49884 strain by another laboratory also displayed a cytosine residue rather than a thymine residue (C. O'Connell, Life Technologies, personal communication). We have deposited our *rpoB *sequence of *M. avium *subsp. *silvaticum *ATCC 49884 in GenBank [GenBank: JN935808].

### Use of *rpoB *sequencing to identify *Mycobacterium *spp. in selected cohorts of animals

In order to facilitate understanding of the mycobacterial diversity in given host groups, we have chosen to present phylogenetic data documenting the use of the *rpoB *sequence to assist with the characterization of *Mycobacterium *spp. in representative cohorts of animals. Note that, to keep our phylogenetic trees to a manageable size, all 386 isolates are not presented in the trees; rather, we focused on incorporating those isolates that best represented the breadth of taxa recovered from the animal groups under study.

**Figure 1:**
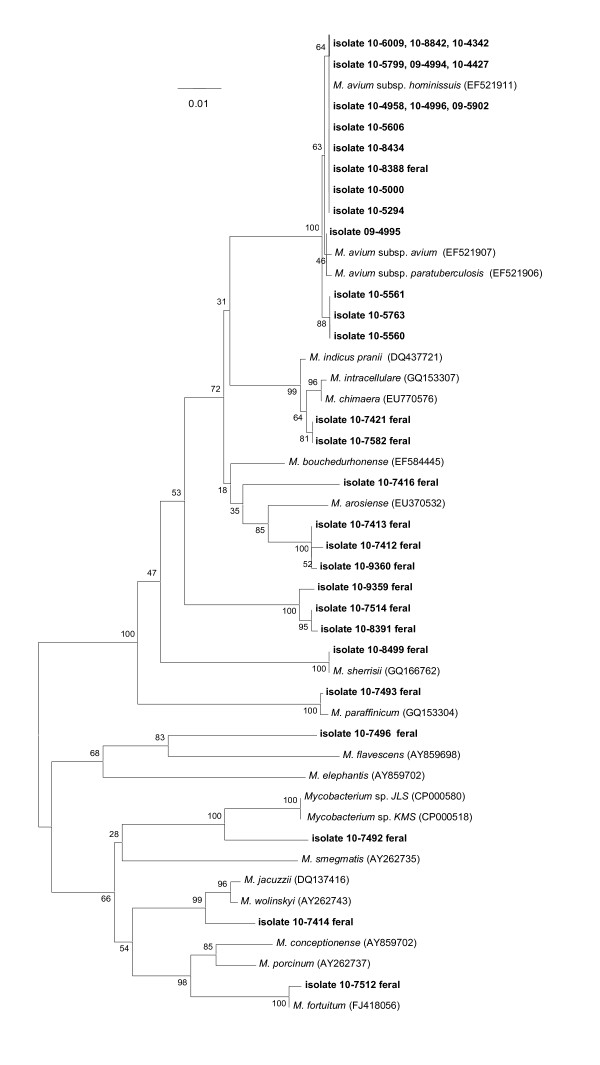
**Neighbor-joining tree generated from a 726-bp sequence of the *rpoB *gene from *Mycobacterium *isolates from 33 domestic and feral pigs and selected reference strains**. Bootstrap values (as a percentage of 1000 replicates) are indicated at nodes. Scale bar indicates evolutionary distance in base substitutions per site.

Figure 1 shows the identities of *Mycobacterium *spp. recovered from the lymph nodes (head, thorax, or abdomen) of 33 domestic and feral pigs in the U.S. Interestingly, all isolates recovered from domestic pigs (n = 19) demonstrated ≥ 99% similarity with MAC, with n = 16 of these showing 100% similarity to *M. avium *subsp. *hominissuis*. In contrast, only one (isolate 10-8388) of 16 isolates obtained from feral swine (trapped in Brooks, Duval, Kenedy, Maverick, and Zapata Counties, Texas) displayed 100% similarity with *M. avium *subsp. *hominissuis*. The remaining 15 feral swine isolates showed considerable diversity in terms of identities with *Mycobacterium *spp. in GenBank. *M. arosiense *represented the most closely related taxa (Figure 1) for three isolates (96% similarity with Nos. 10-7413, 10-7412, and 10-9360), while other feral pig isolates clustered with *M. sherrissii *(100% with 10-8499), *M. paraffinicum *(99% with 10-7493) and *M. fortuitum *(99% with 10-7512). The remaining feral pig isolates displayed ≤ 97% similarity with existing *Mycobacterium *depositions in GenBank, clustering with as-yet unnamed (JLS, KMS), and named (*M. wolinskyi */*M. jacuzzi*), environmental species and/or opportunistic pathogens [16,17].

**Figure 2:**
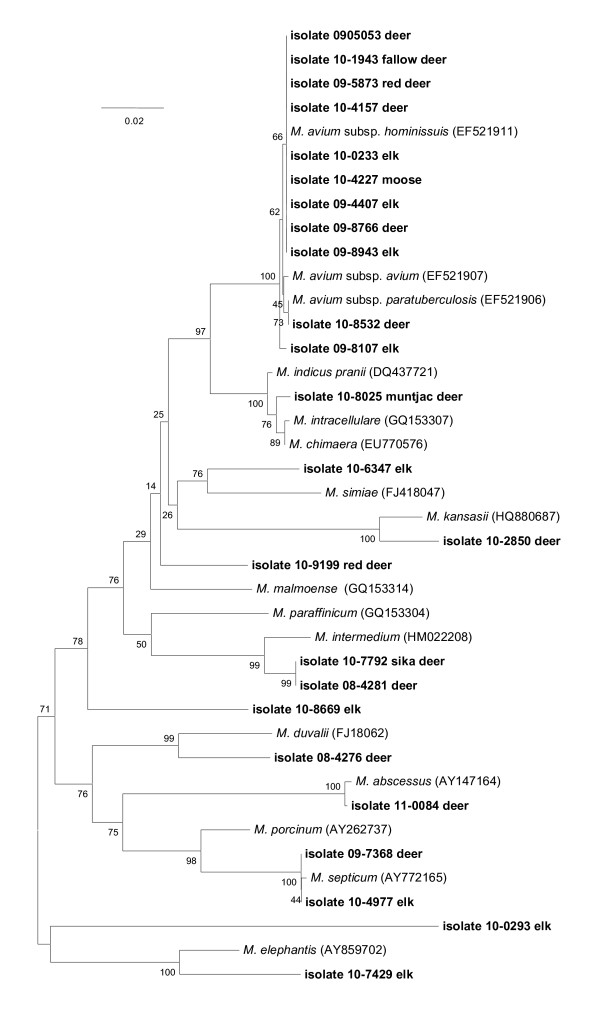
**Neighbor-joining tree generated from a 722-bp sequence of the *rpoB *gene from *Mycobacterium *isolates from 24 cervids and selected reference strains**. Bootstrap values (as a percentage of 1000 replicates) are indicated at nodes. Scale bar indicates evolutionary distance in base substitutions per site.

Figure 2 shows the identities of *Mycobacterium *spp. recovered from the lymph nodes (head, thorax, or abdomen) of 24 cervids (representing deer, moose and elk) in the U.S. Nine isolates displayed 100% similarity with the *rpoB *sequence of *M. avium *subsp. *hominissuis*. Isolates 10-2850 (deer) 10-0293 (elk), 10-8669 (elk), and 10-7429 (elk) displayed < 96% similarity with existing accessions. Other isolates clustered with *M. intermedium *(97% with 10-7792, sika deer, and 08-4281, deer), *M. abscessus *(99% with 11-0084, deer), *M. septicum *(99% with 10-4977, elk, and 09-7368, deer), and the *M. intracellulare *clade (99% with 10-8025, muntjac deer).

**Figure 3:**
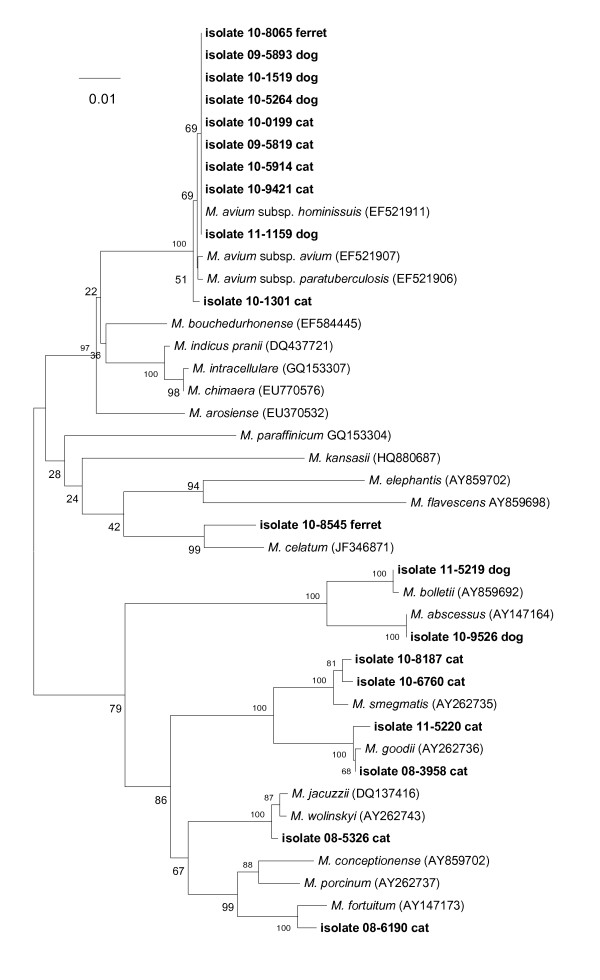
**Neighbor-joining tree generated from a 720-bp sequence of the *rpoB *gene from *Mycobacterium *isolates from 19 companion animals and selected reference strains**. Bootstrap values (as a percentage of 1000 replicates) are indicated at nodes. Scale bar indicates evolutionary distance in base substitutions per site.

Figure 3 shows the identities of 19 *Mycobacterium *spp. recovered from the lymph nodes and skin lesions of companion animals (dogs, cats, and ferrets) in the U.S. Nine (53%) of the isolates possessed an *rpoB *sequence 100% similar to that of *M. avium *subsp. *hominissuis*. Isolate 10-9526 (dog) displayed 100% similarity with the *rpoB *sequence for *M. abscessus*, while two cat isolates (Nos. 10-8187 and 10-6760) segregated at 99% similarity with *M. smegmatis*. Another cat isolate (08-5326) displayed 99% similarity with *M. jacuzzii *and *M. wolinskyi*. A ferret isolate (10-8545) displayed an *rpoB *sequence with 100% similarity to that of *M. celatum*; this species has been documented as a cause of soft tissue infection in ferrets [18]. Isolate 11-5219 from a dog, and isolate 08-3958 from a cat, showed 100% similarity to *M. bolletii *and *M. goodii*, respectively.

**Figure 4:**
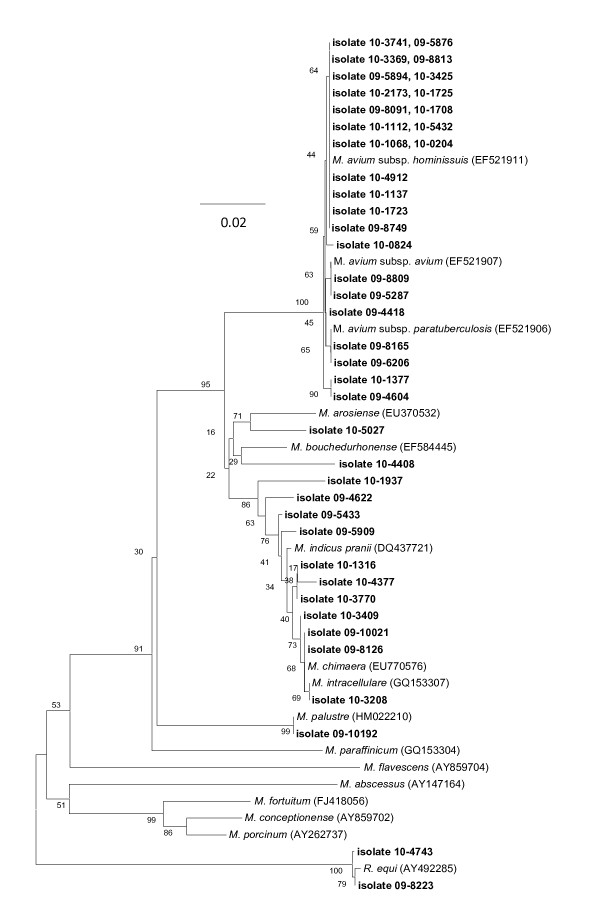
**Neighbor-joining tree generated from a 726-bp sequence of the *rpoB *gene from *Mycobacterium *isolates from 42 cattle and selected reference strains**. Bootstrap values (as a percentage of 1000 replicates) are indicated at nodes. Scale bar indicates evolutionary distance in base substitutions per site.

Figure 4 displays the identities of 42 isolates recovered from the lymph nodes of cattle (dairy and beef) from the U.S. The majority (n = 39) of isolates clustered among the MAC, with 19 (45%) possessing 100% identity with *M. avium *subsp. *hominissuis*. Other of the MAC isolates clustered within the clade containing *M. intracellulare*, *M. indicus pranii*, and *M. chimaera*, with similarities of 99%. One of the non-MAC isolates displayed similarity to *M. palustre *(99% with 09-10192), while the other two (10-4743 and 09-8223) possessed 99% similarity with *Rhodococcus equi*. Indeed, we observed a total of four bovine isolates of putative *Mycobacterium *spp. that were actually *R. equi *by *rpoB *sequencing; to our knowledge, amplification of this species by the Myco-F and Myco-R *rpoB *primers has not previously been reported. In our experience, isolation of this organism from cattle head and thoracic tissue granulomas is not unusual.

**Figure 5:**
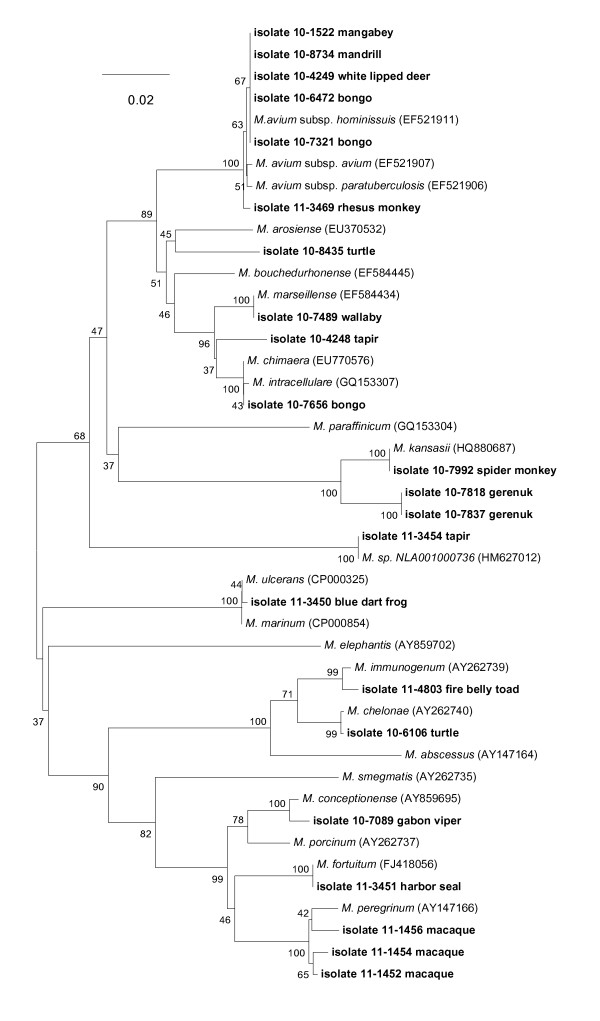
**Neighbor-joining tree generated from a 720-bp sequence of the *rpoB *gene from *Mycobacterium *isolates from 22 zoo animals and selected reference strains**. Bootstrap values (as a percentage of 1000 replicates) are indicated at nodes. Scale bar indicates evolutionary distance in base substitutions per site.

Figure 5 displays the identities of 22 isolates recovered from lymph nodes, oropharyngeal washings, and skin lesions from zoo/exotic animals from the U.S. [The bongo, *Tragelaphus eurycerus eurycerus*, is a large African antelope, while the gerenuk, *Litocranius walleri*, is a variety of East African gazelle]. As with the other cohorts in our study, *M. avium *subsp. *hominissuis *constituted the most frequently isolated species (n = 5 isolates, or 22%). One isolate displayed ≥ 99% similarity with the *rpoB *sequence for *M. marseillense*, a strain recovered from a wallaby (10-7489). Interestingly, two gerenuks from zoos in Florida and Missouri both provided cultures (10-7818 and 10-7837) which displayed < 97% similarity with any reference species. An isolate from a spider monkey (10-7992) from a zoo in Texas displayed 100% similarity with *M. kansasii*, while an isolate from a tapir showed closest similarity (100%) with an unnamed *Mycobacterium *species (NLA001000736) identified in sputum from a patient in Uruguay.

For reptile/amphibian isolates, one from a snake (Gabon viper, 10-7089) displayed 99% similarity with *M. conceptionense*; a fire belly toad isolate (11-4803) displayed 99% similarity with the *M. immunogenum *cluster, while a blue dart frog isolate (11-3450) displayed 99% similarity to the *rpoB *sequence from *M. ulcerans *and *M. marinum*.

Space considerations precluded us from including a figure or table devoted to isolates from elephant tissues and trunk washes, however, we noted that *rpoB *sequencing of 17 such isolates identified members of the MAC, *M. septicum*, *M. holsaticum*, and *M. arosiense *as closest matches to recovered strains of *Mycobacterium*.

### Characterization of novel isolates in the *M. avium *subsp. *paratuberculosis *clade

**Figure 6:**
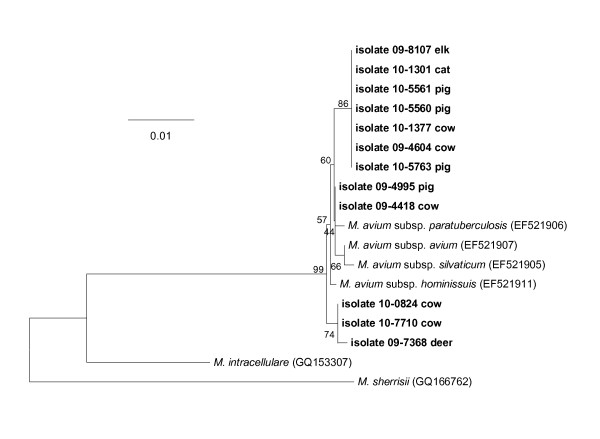
**Neighbor-joining tree generated from a 711-bp sequence of the *rpoB *gene from 12 animal *Mycobacterium *isolates clustering within the sub-clade containing *M. avium *subsp. *paratuberculosis***. Bootstrap values (as a percentage of 1000 replicates) are indicated at nodes. Scale bar indicates evolutionary distance in base substitutions per site.

Over the course of the project we identified 12 isolates which clustered in the clade containing *M. avium *subsp. *avium*, *M. avium *subsp. *paratuberculosis, M. avium *subsp. *silvaticum *and *M. avium *subsp. *hominissuis*, while not displaying 100% identity with the *rpoB *sequence for any of these taxa (Figure 6). The host animals included cattle, a deer, an elk, pigs, and a cat, and were cultured from submissions made during 2009 - 2010. All evidenced ≥ 99.3% similarity with the abovementioned members of this MAC 'sub-clade' (Table 1); all possessed nucleotide substitutions in the third position of the codon, and all possessed satisfactory peaks for their electropherograms, suggesting that the substitutions were not sequencing artifacts.

**Table 1:** Sequence similarity values for a 711 bp portion of the *rpoB *gene from 12 veterinary isolates clustering within the clade containing *M. avium *subsp. *paratuberculosis*, *M. avium *subsp. *avium*, *M. avium *subsp. *hominissuis*, and *M. avium *subsp. *silvaticum*

MAC species	Sequence similarity (%)															
	**1**	**2**	**3**	**4**	**5**	**6**	**7**	**8**	**9**	**10**	**11**	**12**	**13**	**14**	**15**	**16**
**1. pig 10-5763**		99.4	100	100	100	99.7	99.7	100	100	99.3	100	99.4	99.6	99.6	99.4	99.6
**2. cow 10-7710**			99.4	99.4	99.4	99.7	99.7	99.4	99.4	99.9	99.4	100	99.6	99.6	99.4	99.7
**3. pig 10-5560**				100	100	99.7	99.7	100	100	99.3	100	99.4	99.6	99.6	99.4	99.6
**4. pig 10-5561**					100	99.7	99.7	100	100	99.3	100	99.4	99.6	99.6	99.4	99.6
**5. elk 09-8107**						99.7	99.7	100	100	99.3	100	99.4	99.6	99.6	99.4	99.6
**6. cow 09-4418**							100	99.7	99.7	99.6	99.7	99.7	99.9	99.9	99.7	99.9
**7. pig 09-4995**								99.7	99.7	99.6	99.7	99.7	99.9	99.9	99.7	99.9
**8. cow 10-1377**									100	99.3	100	99.4	99.6	99.6	99.4	99.6
**9. cow 09-4604**										99.3	100	99.4	99.6	99.6	99.4	99.6
**10. deer 09-7368**											99.3	99.9	99.4	99.4	99.3	99.6
**11. cat 10-1301**												99.4	99.6	99.6	99.4	99.6
**12. cow 10-0824**													99.6	99.6	99.4	99.7
**13. *M. a. a***.														99.7	99.9	99.7
**14. *M. a. p***.															99.6	99.7
**15. *M. a. s***.																99.6
**16. *M. a. h***.																

*M.a.a*: *M. avium *subsp. *avium*; *M.a.p*.: *M. avium *subsp. *paratuberculosis*; *M.a.s*.: *M. avium *subsp. *silvaticum*; *M.a.h*.: *M. avium *subsp. *hominissuis*. Note that the sequence for *M. avium *subsp. *silvaticum *used in this analysis is Genbank Accession EF521905

These novel MAC isolates were queried via PCR for the insertion elements IS*900 *(considered to be diagnostic for *M. avium *subsp. *paratuberculosis*), IS*901 *(considered to be diagnostic for *M. avium *subsp. *avium*), IS*1245 *(considered to be diagnostic for a number of MAC subspecies), and DT1 (considered to be diagnostic for *M. avium *subsp. *avium *and *M. intracellulare*) [19-21]. Results of these PCR assays are provided in Table 2. All isolates were IS*900 *negative and DT1 negative, and all but one isolate (No. 10-0824, cow) were positive for both IS*1245 *PCR assays.

**Table 2:** Results of PCR assays for five insertion elements for 12 veterinary isolates clustering within the clade containing *M. avium *subsp. *paratuberculosis*, *M. avium *subsp. *avium*, *M. avium *subsp. *hominissuis*, and *M. avium *subsp. *silvaticum*.

Isolate ID	Isolate source	*IS900*	*IS901*	DT1	IS1245 * (long)	IS1245 ** (short)
10-5560	pig	neg	neg	neg	pos	pos
10-5561	pig	neg	neg	neg	pos	pos
10-5763	pig	neg	neg	neg	pos	pos
09-8107	elk	neg	neg	neg	pos	pos
10-7710	cow	neg	neg	neg	pos	pos
09-4418	cow	neg	neg	neg	pos	pos
09-4995	pig	neg	neg	neg	pos	pos
10-1377	cow	neg	neg	neg	pos	pos
09-4604	cow	neg	neg	neg	pos	pos
09-7368	deer	neg	neg	neg	pos	pos
10-1301	cat	neg	neg	neg	pos	pos
10-0824	cow	neg	pos	neg	neg	neg
*M. a. a*.		neg	pos	pos	pos	pos
*M. a. h*.		neg	neg	neg	pos	pos
*M. a. p*.		pos	neg	neg	neg	neg
*M intra*.		neg	neg	pos	neg	neg

* 'long' primers from Van Soolingen et al. (33) ** 'short' primers from Johansen et al. (20)

neg = negative, pos = positive

*M.a.a*: *M. avium *subsp. *avium*; *M.a.h*.: *M. avium *subsp. *hominissuis*; *M.a.p*.: *M. avium *subsp. *paratuberculosis*; *M. intra*.: *M. intracellulare*

Interestingly, this isolate, No. 10-0824, was positive for the IS*901 *element. BLAST analysis of 733 nucleotides sequenced from this PCR product showed 99% similarity with [Genbank: AB447556], the *M. avium *subsp. *hominissuis *ISMav6 gene, and 96% similarity with [GenBank: X58030] and [GenBank: AF527973], the *M. avium *subsp. *avium *insertion elements 902 and 901, respectively. Although this same isolate was negative for both IS*1245 *PCR assays, it should be noted that the absence of this element has been documented in some isolates of *M. avium *subsp. *avium *[22].

## Discussion

In our hands, the *rpoB *PCR assay readily generated amplicons from > 99% of isolates tested, and produced quality sequences for > 95% of those isolates.

When both 16S rRNA and *rpo*B sequencing were performed on a subset of 236 isolates, the percentage of identities matching at the species or clade level with both methods was 70%, a figure lower than that (86%) reported by Simmon et al. [14] in their combined sequencing analysis of clinical isolates. The greater degree of discrepancy in our panel may be attributed to the dearth of longer-length (i.e., ≥ 711 bp) *rpoB *sequences in GenBank for type specimens of *M. bohemicum*, *M. asiaticum*, *M. obuense*, etc. As well, we obtain 16S rRNA identities for our *Mycobacterium *isolates using the Ridom database, which compares ≤ 450 bp of sequence; use of this smaller segment of the 16S rRNA gene may contribute to discrepant results between the assignations with 16S rRNA and *rpoB*. We have noticed that when we use GenBank to identify species, using the longer-length reads (≥ 600 bp) obtained with our 16S rRNA sequences, the resulting species identity is sometimes more likely than the Ridom assignation to match the species identity obtained with the *rpoB *sequence. However, the Ridom database is curated, unlike GenBank, and thus results obtained with the former database are preferable from the standpoint of adhering to the ISO17025 standards governing the operation of our laboratory.

Additionally, some of the isolates for which *rpoB *similarities were < 98% with reference sequences may (arguably) represent new species. We note that anecdotally, the descriptions of new species of veterinary origin have tended to lag behind those of human origin, which may contribute to the ambiguity surrounding assignations of identities to some of the isolates in our panel. As we expand the use of *rpoB *sequencing in our laboratory, we intend to continue adding sequences of ≥ 711 bp from (currently unrepresented) species and veterinary isolates to GenBank, as this will improve the ability of this locus, and assays derived from it, such as the RipSeq assay [14], to identify mycobacterial species.

A weakness of the combined use of 16S rRNA and *rpoB *sequences for species assignation is that some clades of *Mycobacterium *may not be partitioned to species level via the combined use of these two loci. For example, Zelazny et al. [23] examined 42 clinical isolates belonging to the *M. abscessus *group and observed that *rpoB *sequencing parsed this collection into 33 *M. abscessus *isolates, 7 *M. massiliense *isolates, and 2 *M. bolletii *isolates. The inclusion of additional sequence data from the *secA *and *hsp65 *genes resulted in the assignation of 26 *M. abscessus *isolates, 7 *M. massiliense *isolates, and 2 *M. bolletii *isolates; the remaining 7 isolates ultimately were assigned to *M. massiliense *via use of ITS sequencing. While the use of multilocus sequence typing (MLST) incorporating loci such as *hsp65 *and *secA *undoubtedly would aid in the more accurate identification of isolates, the current economics of veterinary diagnostic testing rule against routine use of MLST in our laboratory.

Our evaluation of *rpoB *sequencing indicated that it will be of particular value in assigning species designations to members of the MAC. Of the 386 isolates for which *rpoB *sequence was obtained, 184 (47%) were members of the MAC, and the most frequently isolated species was *M. hominissuis *(60 isolates, 15%). Similar to the results of Ben Salah et al. [13], who observed that *M. avium *subsp. *hominissuis *constituted the major MAC member in their panel of 100 clinical isolates, this subspecies was the predominant MAC isolated from all animals in our study. The cosmopolitan distribution of *M. avium *subsp. *hominissuis *observed among our panel of host animals suggests that this subspecies may be responsible for a substantial proportion of mycobacterial disease among livestock, companion animals, and zoo/exotic animals.

For feral swine isolates, non-MAC species dominated the assemblage, including novel environmental species such as *Mycobacterium *sp. JLS/KMS, suggesting that these animals are exposed to a variety of mycobacterial taxa in the course of foraging for food in the forest litter and pasture land. Also noteworthy is the observation that 8 feral pig isolates displayed < 97% similarity with existing GenBank accessions, suggesting that some of these isolates may represent new species. The implications for transmission of these mycobacteria to cattle or domestic pigs, with whom feral pigs often come into contact, are unclear, but in light of the fact that feral swine may harbor important veterinary pathogens (such as *Brucella *spp.), further investigation may be warranted [24,25].

*M. marseillense*, *M. timonense*, and *M. bouchedurhonense *are recently described members of the MAC; all three species were originally recovered from patients in southern France. In their survey of 139 clinical isolates of U.S. origin, Simmon et al. [14] observed two isolates with *rpoB *sequence similarity to that of *M. timonense *and *M. bouchedurhonense*. We observed two elephant and one feral pig (all three animals located in Texas) isolates with 96 - 97% similarity to the *rpoB *sequence for *M. bouchedurhonense*. Isolates from an elephant (eastern United States), a wallaby (location data not available), and a domestic cat (location unavailable) displayed 99 - 100% similarity to *M. marseillense*. While the precise location at which the infection was acquired by the host animal obviously cannot be determined with certainty, these findings do expand the categories of host animal and geographic locales for these species.

For other members of the MAC, a number of cattle, pig, and cervid isolates segregated into the clade containing *M. chimaera *and *M. intracellulare*. However, we did not observe any isolates with *rpoB *sequences with > 98% similarity with *M. colombiense*. Regarding *M. avium *subsp. *silvaticum*, the latter species has not been encountered in diagnostic submissions to the NVSL, suggesting that it does not represent a significant source of morbidity or mortality in some groups of animals and birds. However, we have no record of receiving wood pigeons over the past decade, during which we received 100 - 200 avian specimens each year. Our analysis of *M. avium *subsp. *silvaticum *indicates that, at least for the ATCC 49884 strain, differentiating it from *M. avium *subsp. *avium *may not be possible based on the *rpoB *sequence amplified with the Myco-F and Myco-R primer set.

*M. avium *subsp. *silvaticum *reportedly has a variable biochemical requirement for mycobactin J, consequently NVSL uses a media protocol involving formulations with and without mycobactin J for all avian submissions, so dependency on this reagent would be recognized. Accordingly, despite the identical *rpoB *sequence for *M. avium *subsp. *silvaticum *and *M. avium *subsp. *avium*, we have some degree of confidence in our ability to recognize *M. avium *subsp. *silvaticum *in our laboratory. We anticipate that the phylogenetic and taxonomic status of *M. avium *subsp. *silvaticum *ATCC 49884, as a member of the MAC, will be clarified by ongoing genomic sequencing efforts (C. O'Connell, Life Technologies, personal communication).

We identified 12 isolates from cattle, cervids, pigs, and a cat that clustered with the clade containing *M. avium *subsp. *avium*, *M. avium *subsp. *silvaticum*, *M. avium *subsp. *paratuberculosis*, and *M. avium *subsp. *hominissuis*; however, these isolates did not display 100% similarity with any of these subspecies.

The implications of this observation for diagnostic purposes are unclear. *M. avium *subsp. *avium *and *M. avium *subsp. *paratuberculosis *are important livestock and companion animal pathogens that are tested for on a routine basis by veterinary diagnostic laboratories, using commercial real-time and conventional PCR assays designed to target insertion elements considered to be unique to the individual subspecies [26]. Our evaluation of the insertion element profiles (i.e., IS*900*, IS*901*, DT1, and IS*1245*) for these novel MAC isolates indicates that they display some heterogeneity in this regard, making assignation to a defined subspecies problematic. We are pursuing more detailed genetic analyses of these isolates (i.e., genomic sequencing) in order to better characterize their taxonomic position within the MAC.

Currently, standard operating procedures in our laboratory for identification of rapid- and slow- growing *Mycobacterium *involve selected Gen-probe^® ^assays, in conjunction with traditional biochemical tests and 16S rRNA sequencing. Given that 16S rRNA sequences are identical among *M. avium *subsp. *avium*, *M. avium *subsp. *paratuberculosis, M. avium *subsp. *silvaticum*, and *M. avium *subsp. *hominissuis *, resolution to the subspecies level has not been feasible, and thus cases associated with these agents historically have been reported to clients as '*M. avium *complex'. Our study demonstrates the utility of *rpoB *PCR and sequencing for identification of subspecies within the MAC, and other species of mycobacteria, derived from veterinary specimens.

## Conclusions

Our panel of 386 veterinary isolates represents the most comprehensive such collection yet used to evaluate *rpoB *sequencing as a method of identification to species and/or clade level. Our study confirms observations made from studies performed on human isolates, namely, that *rpoB *sequencing can aid in the identification of *Mycobacterium *spp. to the clade or species level. This is particularly true for species belonging to the MAC, which continues to represent a major lineage associated with infections in a variety of livestock and companion animals. We note the some mycobacterial assemblages, as the *M. abscessus *group, require additional loci/MLST for adequate resolution, as a combination of 16S rRNA and *rpoB *sequencing alone is inadequate to assign a species identity. Accordingly, the use of 16S rRNA and *rpoB *sequencing necessarily presents with some limitations. However, in the current context of veterinary diagnostics, the use of 16S rRNA and *rpoB *sequencing represents an affordable and cost-effective paradigm for species identification, particularly when compared to methods such as culture- and biochemical- based assays.

## Methods

### Mycobacterial strains

Isolates were cultured from specimens submitted to the Mycobacteria and Brucella Section, NVSL in Ames, Iowa, during 2008 - May 2011. Specimens included tissue samples, which were externally decontaminated using a sodium hypochlorite/sodium hydroxide-based procedure [27], oropharyngeal swabs, and swabs of skin lesions. Media used for isolation included customized formulations made at the NVSL: 7H11 agar with hemolyzed blood, serum, OADC (oleic acid-albumin-dextrose- catalase) and pyruvate; 7H10 agar with OADC and pyruvate; Stonebrinks media; Lowenstine-Jensen media (Becton Dickson, Sparks, Maryland, USA), Bactec 460 and Bactec MGIT 960 liquid culture media (Becton Dickson, Sparks, Maryland, USA); Herrold egg yolk agar, and the ESP II liquid culture system (TREK Diagnostic Systems, Cleveland, OH) [28].

Genomic DNA was extracted by placing a loopful of cells, or the cell pellet derived from ~ 100 μl of turbid liquid media, into a 2.0 ml tube containing 300 μl phenol-chloroform-isoamyl alcohol, 300 μl Tris-EDTA buffer, and 200 μl 0.1 mm silica beads. The contents were mixed and the tube subjected to rotation on a specialized instrument (Mini-Beadbeater, BioSpec Products, Inc., Bartlesville, OK, USA) at maximum setting for 90 sec. Following lysis, the bead tube was centrifuged at 16, 000 × *g *for 5 min and the supernatant removed and transferred to a 1.5 ml centrifuge tube and the DNA precipitated using ethanol and 3M sodium acetate. DNA was reconstituted in 300 μl TE buffer, or molecular biology grade water, and stored at -70°C, with 2 μl used as template for PCR (below). Extraction controls, consisting of sterile water, were included with all extraction procedures.

### PCR and sequencing

For 16S rRNA PCR and sequencing, genomic DNA was amplified using primer 27 (forward) and 907 (reverse) which amplify a ~ 909 bp portion of the 16s rRNA gene [29]. Initial amplification conditions were: 95°C for 5 min, followed by 30 cycles of 94°C for 45 sec, 53°C for 60 sec, and 72°C for 90 sec and a single 10 min elongation step at 72°C. Negative PCR controls included reactions containing 5 μl water as template. The PCR amplicons were treated with ExoSap-IT (USB/Affymetrix, Santa Clara, CA, USA) and subjected to dye terminator cycle sequencing using two primer pairs, 271 forward and 519 reverse; and 27 forward and 519 reverse [29,30]. Sequencing products were treated with BigDye XTerminator purification Kit (Applied Biosystems, Foster City, CA) and electrophoresed on an Applied Biosystems 3500XL genetic analyzer. Consensus sequences (constituting ~ 450 bp at the 5' end of the 16S rRNA gene) were assembled using Lasergene^® ^software (DNASTAR, Madison, WI) and submitted to the Ridom 16S rDNA website http://www.ridom.com. Assignation to species level was done for sequences with similarities of ≥ 99% with entries in the Ridom database, while sequences with < 99% similarity were assigned complex (i.e., MAC) or clade-level identities.

For *rpoB *PCR and sequencing, the protocol of Ben Salah et al. [13] was used. Briefly, genomic DNA was amplified using the Myco-F (5' GGCAAGGTCACCCCGAAGGG 3'; base positions 2479-2498 with reference to the *M. paratuberculosis *K-10 rpoB sequence) and Myco-R (5' AGCGGCTGCTGGGTGATCATC 3'; base positions 3219-3239) primers, which amplify a ~ 760 bp portion of the *rpoB *gene. PCR reactions consisted of 3 - 5 μl (equivalent to 10 - 20 ng) DNA, 5 μl 10× buffer and 1 *U Taq *polymerase (AmpliTaq^® ^Gold, Applied Biosystems, Foster City, CA), 2.5 μl 25 mM MgCl_2_, and 50 pmol each primer, in a total volume of 50 μl. Sequencing reaction conditions were the same as those described above for 16S rRNA sequencing. Resultant *rpoB *sequences (~ 730 bp) were analyzed using MEGA version 5.0 software [31]. Neighbor-joining trees were constructed from 1000 bootstrap replicates of each alignment from distances estimated using the Jukes-Cantor method as implemented in MEGA, with values > 75% considered to be significant. As per the recommendation of Adekambi et al. [11,12] sequences with similarities of ≥ 98% with reference sequences in GenBank were identified to species. Sequences at < 98% similarity were identified to clade level (for example, the *M. intracellulare */*M. chimaera */*M. indicus pranii *clade) based on their placement with reference sequences in the neighbor-joining tree.

Differentiation among members of the MAC clade containing *M. avium *subsp. *avium*, *M. avium *subsp. *paratuberculosis, M. avium *subsp. *hominissuis*, and *M. avium *subsp. *silvaticum *was done on the presence of the following nucleotide polymorphisms (using the numbering scheme for the *M. avium *subsp. *paratuberculosis *K10 strain *rpoB *gene [GenBank: NC_002944] [32]: nucleotide 2, 541: T/C for *M. avium *subsp. *silvaticum*, C for the other three subspecies; nucleotide 2, 724: T for *M. avium *subsp. *hominissuis*, C for the other three subspecies; nucleotide 2, 790: A for *M. avium *subsp. *paratuberculosis*, G for the other three subspecies; nucleotide 2, 898: C for *M. avium *subsp. *hominissuis *and *M. avium *subsp. *paratuberculosis*, G for *M. avium *subsp. *avium *and *M. avium *subsp. *silvaticum*.

### Insertion element PCR

Two sets of primers were used for the IS*1245 *insertion element: the 'long' P1 and P2 primers which amplify a 427 bp fragment [33], and the 'short' P40 and P41 primers which amplify a 175 bp fragment [20]. Neither the long primer pair nor the short primer pair have been reported to amplify *M. avium *subsp. *paratuberculosis*, although the short primer pair will amplify *M. avium *subsp. *hominissuis*, *M. avium *subsp. *avium *and *M. avium *subsp. *silvaticum *[20] (M. Pateja, unpublished data). The protocol of Kim et al. [26], using the P2 probe and the F3/R3 primer set, was used for the IS*900 *PCR; this insertion element is considered to be unique to *M. avium *subsp. *paratuberculosis*. The IS*901 *and DT1 PCR assays used the primers of Shin et al. [21]; the former insertion element is considered to be unique to *M. avium *subsp. *avium*, while the latter element is present in both *M. avium *subsp. *avium *and *M. intracellulare*. Insertion element PCR reactions and thermal cycling conditions were performed according to the protocols provided in the above publications.

### Internal validation of *rpoB *sequencing

A panel of 14 *M. avium *subsp. *hominissuis *isolates (8 isolated from pigs and 6 from humans) of Central European origin was used as an external validation of the *rpoB *sequencing assay. These isolates had been identified on the basis of biochemical characteristics, IS*1245 *RFLP, IS*901 *PCR, and MIRU/VNTR [34,35]. All 14 isolates generated *rpoB *sequences that were 100% similar to the GenBank reference sequence for *M. avium *subsp. *hominissuis *[GenBank: EF521911].

To confirm the stability of the nucleotide polymorphisms in the *rpoB *sequence used to assign species identity, an isolate of *M. paratuberculosis *K10 strain that had been cultured for 8 consecutive passages was subjected to DNA extraction and *rpoB *sequencing for each of the passages. All passages displayed 100% similarity with the reference sequence for *M. avium *subsp. *paratuberculosis *[GenBank: EF521906].

To confirm the reproducibility of sequences generated from given isolates using different instruments, different lots of reagents, and different aliquots of genomic DNA, a panel of 7 *M. avium *subsp. *hominissuis*, one *M. avium *subsp. *paratuberculosis*, one *M. avium *subsp. *avium*, and one *M. conceptionense *isolate were sequenced using an ABI 3130 instrument, and then 4 - 8 weeks later, sequenced again using an ABI 3500 XL instrument. All 10 isolates displayed 100% similarity between the first and second replicates.

### Deposition of nucleic acid sequences

The following *rpoB *gene DNA sequences (710 - 750 bp) have been deposited in GenBank and assigned accession numbers: *Mycobacterium kansasii *ATCC 12478 [GenBank: HQ880687]; *M. celatum *ATCC 51130 [GenBank: JF346871]; *M. abscessus *ATCC 19977 [GenBank: JF346872]; *M. gordonae *ATCC 14470 [GenBank: JF346873]; *M. fortuitum *ATCC 6841 [GenBank: JF346874]; *M. smegmatis *ATCC 35797 [GenBank: JF346875]; *M. terrae *ATCC 15755 [GenBank: JF346876]; *M. triviale *ATCC 23290 [GenBank: JF712873]; *M. intermedium *ATCC51848 [GenBank: JF712874]; *M. neoaurum *ATCC 25795 [GenBank: JF712875]; *M. peregrinum *ATCC 14467 [GenBank: JF712876]; *M. flavescens *ATCC 14474 [GenBank: JF712877]; *M. mageritense *ATCC 700351 [GenBank: JF706630]; *M. senegalense *ATCC 35796 [GenBank: JF706631]; *M. avium *subsp. *silvaticum *ATCC 49884 [GenBank: JN935808]; *M. szulgai *ATCC 29716 [GenBank: JN881348]; *M. xenopi *ATCC 19972 [GenBank: JN881349]; *M. lentiflavum *ATCC 51985 [GenBank: JN881350]; *M. nonchromogenicum *ATCC 19530 [GenBank: JN881351]; *M. gastri *ATCC 15754 [GenBank: JN986748]; and the following veterinary clinical isolates: domestic pig [GenBank: JF327744]; domestic steer [GenBank: JF327745]; bovine [GenBank: JF437543]; bovine [GenBank: JF437544]; domestic pig [GenBank: JF437545]; white-tail deer [GenBank: JF437546]; domestic cat [GenBank: JF437547]; elk [GenBank: JF437548]; domestic pig [GenBank: JF437549]; bovine [GenBank: JF437550]; bovine [GenBank: JF437551]; domestic pig [GenBank: JF437552]; Rhesus macaque [GenBank: JF804804].

## Authors' contributions

JH designed the study, implemented the methods, analyzed sequence data, and wrote the manuscript. PC, DF, DB, and MP extracted samples, cultured *Mycobacterium *spp., performed PCR and sequencing assays, conducted BLAST and Ridom analyses, managed the sequencing databases, and contributed the Tables to the manuscript. SRA processed samples and cultured *Mycobacterium*, supervised bench work associated with the project, and contributed to the manuscript. All authors have reviewed and approved this manuscript.

## Supplementary Material

Additional file 1 ***Mycobacterium *spp. isolates subjected to sequencing**. A pdf file of an Excel sheet listing 236 isolates of *Mycobacterium *of veterinary origin for which both 16S rRNA and *rpoB *sequencing was performed. For each isolate, the clade, species, or subspecies identity assigned by 16S rRNA sequence (made using the Ridom website, http://rdna.ridom.de/), and the identity assigned by *rpoB *sequence (made using NCBI BLAST comparisons), are provided. Click here for file

Additional file 2 **Presence of a cytosine residue in the *rpoB *sequence of *M. avium *subsp. *silvaticum *ATCC 49884**. A pdf file of a ABI 3500XL electropherogram depicting the base calls for the region of the *rpoB *gene of *M. avium *subsp. *silvaticum *ATCC 49884 where nucleotide 2, 541 (using the *M. avium *subspecies *paratuberculosis *rpoB K10 strain numbering convention) presents as a cytosine (outlined in the yellow box). Click here for file
