# Supplementary material for: Identification of Mycobacterium spp. of veterinary importance using rpoB gene sequencing
Source: BMC Vet Res. 2011 Nov 25;7:77. doi: 10.1186/1746-6148-7-77 (PMC3251535; doi:10.1186/1746-6148-7-77)
Supplement: Additional file 1 — Mycobacterium spp. isolates subjected to sequencing. A pdf file of an Excel sheet listing 236 isolates of Mycobacterium of veterinary origin for which both 16S rRNA and rpoB sequencing was performed. For each isolate, the clade, species, or subspecies identity assigned by 16S rRNA sequence (made using the Ridom website, http://rdna.ridom.de/), and the identity assigned by rpoB sequence (made using NCBI BLAST comparisons), are provided. [file 1746-6148-7-77-S1.PDF]

Case No.	Isolate info	Ridom 16S rRNA sequencing call*	*rpoB* sequence call	Date	Match code (yes = 1, no = 0)
08 3958	cat	Ridom = M. goodii 100%	BLAST = 99% with M. goodii (AY262736)	Nov 26 2010	1
08 4276	deer	Ridom = M. duvalii 100%	BLAST = 94% with M. duvalii (FJ418062)	Nov 26 2010	1
08 4281	deer	Ridom = M. intermedium 100%	BLAST = 97 % with M. intermedium (HM022208)	Nov 26 2010	1
08 5326	cat	Ridom = M. wolinsky 100%	BLAST = M. jacuzzii (99% with DQ137416), M. wolinskyi (99% with AY262743)	Dec 2 2010	1
08 5799	raccoon	Ridom = MAC 100%	BLAST / MEGA align = M hominissuis (100% with EF521911)	Nov 26 2010	1
08 6190	cat	Ridom = M. fortuitum 99%	BLAST = 97 % with M. fortuitum (EU770579)	Nov 26 2010	1
10 5946	cow	Ridom 16S = 100 % M. porcinum / fortuitum	M. porcinum (100 % with AY262737)	"	1
10 4841	cichlid fish	Ridom 16S = M. simiae 100%	BLAST = many hits at 94 %; MEGA align = M. pulveris ?	"	0
10 5197	cow	Ridom 16S = M. pulveris 99%	M. pulveris (99% with AY859701)	"	1
10 4543	elephant	Ridom 16S = M. intracellulare 99%	BLAST = many hits at 96, 95 %; MEGA align = unique clade ?	"	0
10 5181	cow	multiple hits, M. szulgai / kansasii highest scores at 99 %	BLAST = M. intermedium (97 % with HM022208) (checked again on 5-3-2011 JH)	June 10 2010	0
10 5355	elephant	Ridom 16S =100 % M. intracellulare	BLAST = M. chimaera (99% with EU770576) & M. intracellulare (99% with GQ153307)	"	1
10 5357	elephant	Ridom 16S =100 % M. intracellulare	BLAST = M. intracellulare (100 % with GQ153307, 99% M. chimaera (99% with EU770576)	"	1
10 5527	elephant	Ridom 16S =100 % M. intracellulare	BLAST = M. intracellulare (100 % with GQ153307, 99% M. chimaera (99% with EU770576)	June 10 2010	1
10 5658	cow	Ridom 16S = 100% MAC	BLAST = M. hominissuis (100% with EF521911), M. avium (99% with GQ153306)	"	1
10 5094 rough	cow	Ridom 16S = M. septicum / M. peregrinum 99%	BLAST = M. peregrinum (100 % with AY147166)	June 17, 2010	1
10 5094 smooth	cow	Ridom 16S = M. septicum / M. peregrinum 99%	BLAST = M. peregrinum (100 % with AY147166)	"	1

10 5941	reference culture	Ridom 16S = M. kansasii / M. gastri 99%	BLAST = M. kansasii (100 % with EU370227) (99% with our HQ880687, 5-3-2011, JH)	"	1
10 6106	turtle	Ridom 16S = M. abcessus/ M. chelonae 99%	BLAST = M. chelonae (100 % with AY262740)	"	1
10 6472	bongo (an antelope)	Ridom = MAC 100%	BLAST = M. hominissuis (100% with EF521911), M. avium (99% with GQ153306)	"	1
10 5355	elephant (same as 10 4543)	Ridom = M. intracellulare 100%	BLAST = M. bouchedurhonense (95 % with EF584445), M. vulneris (95 % with EU834057)	July 1 2010	0
10 5357	elephant (same as 10 4543)	Ridom 16S = M. pulveris 100%	BLAST = (M. bouchedurhonense (95 % with EF584445), M. arosiense (94% with EU370532)	"	0
10 5881 gray	cow	Ridom 16S = MAC 100%	BLAST = M. avium 104 (99% with CP000479); MEGA rpoB align = 1 base off from both M. para or M. avium	July 1 2010	1
10 5980	cow	Ridom 16S = M. porcinum / M. fortuitum 100%	BLAST = M. porcinum (99% with AY262737)		1
10 6021	cow	Ridom 16S = MAC 100%	BLAST = 100% with avium and 99% with para; MEGA rpoB align 100% with M. hominissuis	"	1
10 6127	cow	Ridom 16S = M. paraffinicum 99%	BLAST = M. nebraskense (97 % with GQ153310)	"	0
10 6347	elk	Ridom 16S = M. asiaticum 99%	BLAST = 94% with M. montefiorense (HM022209)	Nov 26 2010	0
10 6424	cow	Ridom 16S = M. neoaurum 100%	BLAST = M. neoaurum (99 % with FJ172335)	July 1 2010	1
10 6441	cow	Ridom 16S = M. farcinogenes, M. fortuitum, M. senegalense 99%	BLAST = M. farcinogenes (98% with AY262742)	"	1
10 6510	cow	Ridom 16S = M. smegmatis 100%	BLAST = M. smegmatis (98% with AY262735)	"	1
10 4088	cow	Ridom 16S = M. szulgai 100%	BLAST = M. interjectum (94% with HM022207)	"	0
09 7792	sika deer	16S = M. intermedium 100%	BLAST = M. intermedium (97% with HM022208)	"	1
10 4542	elephant (same as 10 5527)	16S = M. szulgai 99%	BLAST = M. interjectum (94% with HM022207)	"	0
10 5899	cow	BLAST = M. nonchromogenicum 99%	BLAST = 91% with M. scrofulaceum (GQ153305)	July 15 2010	0

10 6228	cow	BLAST = MAC 100%	BLAST = M. hominissuis (100% with M. avium 104, CP000479); MEGA align 100 % with M. hominissuis	"	1
10 6319	cow	BLAST = MAC 100%	BLAST = M. avium (100% with M. avium 104, CP000479); MEGA align 100% with M. hominissuis	"	1
10 6322	cow	BLAST = MAC 100%	BLAST = M. avium (99% with M. avium 104, CP000479); MEGA align 100% with M. hominissuis	"	1
10 6347	elk	Ridom 16S = M. asiaticum 99%	BLAST = M. simiae (93 % with FJ418047)	Sept. 10 2010	0
10 6441	cow	Ridom 16S = M. farcinogenes, M. fortuitum, M. senegalense 99%	BLAST = M. farcinogenes, M. senegalense (98%), M. fortuitum (97%)	August 12, 2010	1
10 6471	elephant	Ridom 16S = M. intracellulare 100%	BLAST = M. marseillense (99% with EF6=584434)	Sept. 3 2010	0
10 6756	cow	BLAST = MAC 100%	BLAST = M. avium (99% with M. avium 104, CP000479); MEGA align 100% with M. hominissuis	July 15 2010	1
10 6760	cat	BLAST = M. smegmatis 99%	BLAST = 99% with M. smegmatis (CP000480)	"	1
10 7216	cow	Ridom 16S = M. lentiflavum 99%	BLAST = M. palustre (100% with HM022210)	August 12, 2010	0
10 7003	cow	BLAST = M. intracellulare 99%	BLAST = 99% with M. indicus pranii (DQ437721), 99% with M. chimaera (EU770576), 99% with M. intracellulare (GQ153307)	August 12, 2010	1
10 7234	cow	BLAST = M. smegmatis 99%	BLAST = 99% with M. smegmatis (CP000480, AY262735)	July 15 2010	1
10 7246	cow	Ridom 16S = M. lentiflavum 99%	BLAST = M. palustre (100% with HM022210)	August 12 2010	0
10 7367	cow	Ridom 16S = M. smegmatis 99%	BLAST = 99% with M. smegmatis (CP000480)	Sept. 3 2010	1
10 7089	gabon viper	Ridom 16S = M. farcinogenes,M.fortuitum,M. senegalense, all 100%	BLAST = 99% with M. conceptionense (AY859695)	August 5 2010	0
10 7089	gabon viper	Ridom 16S = M. farcinogenes,M.fortuitum,M. senegalense, all 100%	BLAST = 99% with M. conceptionense (AY859695)	Sept 16 2010 (retest of same sample)	0
10 7429	elk	Ridom 16S = M. confluentis 96.7%	BLAST = 94% with M. elephantis (AY859702)	August 5 2010	0
10 7251	cow	Ridom 16S = MAC 100%	BLAST / MEGA align = M hominissuis (100% with EF521911)	"	1

10 7257	cow	Ridom 16S = MAC 100%	BLAST / MEGA align = M hominissuis (100% with EF521911)	"	1
10 7273	cow	Ridom 16S = Mtb complex 100%	BLAST = 100% with M. tuberculosis (CP001658) / M. bovis (AP010918)	August 26 2010	1
10 7357	cow	Ridom 16S = MAC 100%	BLAST / MEGA align = M hominissuis (100% with EF521911)	"	1
10 7358	cow	Ridom 16S = MAC 100%	BLAST / MEGA align = M hominissuis (100% with EF521911)	"	1
10 7368	cow	Ridom 16S = M. septicum / M. peregrinum 100%	BLAST =M. septicum (99% with AY772165)	Sept. 10 2010	1
10 7370	cow	Ridom 16S = M. lentiflavum 100%	BLAST = M. palustre (96% with HM022210)	Sept 24 2010	0
10 7434	elephant	Ridom 16S = M. intracellulare 100%	BLAST = 99% with M. indicus pranii (DQ437721), 99% with M. chimaera (EU770576), 99% with M. intracellulare (GQ153307)	August 19 2010	1
10 7447	cow	Ridom 16S = M. smegmatis 100%	BLAST = 99% with M. smegmatis (CP000480, AY262735)	August 19 2010	1
10 7457	cow	Ridom 16S = M. porcinum / M. fortuitum 99%	BLAST = 99% with M. porcinum (AY262737)	"	1
10 7489	wallaby	Ridom 16S = M. intracellulare 100%	BLAST, MEGA = M. marseillence (100% with EF584434)	Sept 30 2010	0
10 7656	bongo	Ridom 16S = M. intracellulare 100%	BLAST = 100% with M. chimaera (EU770576), 99% with M. intracellulare (GQ153307)	August 26 210	1
10 7710	cow	Ridom = MAC 100%	MEGA align = M. hominissuis / M avium / M paratuberculosis (99% with EF521911. EF521907, & EF521906)	Sept 16 2010	1
10 7818	gerenuk	Ridom 16S = M. kansasii, 1 mismatch 99%	BLAST = 97% with M. kansasii (EU370227) and 97% with HQ880687. 5-3-2011 JH	August 19 2010	1
10 7821	cow	Ridom 16S = M. septicum / M. peregrinum 100%	BLAST = M. peregrinum (99 % with AY147166)	"	1
10 7837	gerenuk	Ridom 16S = M. kansasii, 1 mismatch 99%	BLAST = 98% with M. kansasii (HQ880687) 5-3-2011 JH	August 19 2010	1
10 7874	elephant	Ridom = M. terrae 100%	BLAST = 91% with M. malmoense (GQ153314)	Sept 30 2010, Dec 9 2010	0
10 7884	cow	Ridom = M. simiae 100%	BLAST = M. simiae (99 % with FJ418047)	Oct 14 2010	1
10 7992	spider monkey	Ridom 16S = M. kansasii, M. gastrii 99%	BLAST = 100 % with M. kansasii (HQ880687) 5-3-2011 JH	Sept 10 2010	1

10 8025	muntjac deer	Ridom = M. intracellulare 100%	BLAST = 99% with M. indicus pranii (DQ437721), 99% with M. chimaera (EU770576), 99% with M. intracellulare (GQ153307)	Sept 16 2010	1
10 8025 (rep 2)	muntjac deer	Ridom = M. intracellulare 100%	BLAST = 99% with M. indicus pranii (DQ437721), 99% with M. chimaera (EU770576), 99% with M. intracellulare (GQ153307)	Sept 24 2010	1
10 8014	cow	Ridom = M. kansasii, M. szulgi 99%	BLAST = 98% with M. intermedium (HM022208)	Oct 21 2010	0
10 8187	cat	Ridom 16S = M. smegmatis 100%	BLAST = 99% with M. smegmatis (CP000480, AY262735)	Aug 26 2010	1
10 8187 yellow	cat	Ridom 16S = M. smegmatis 100%	BLAST = 100% with M. smegmatis (CP000480, AY262735)	Aug 26 2010	1
10 8231	tanager	Ridom = M. genavense 100%	BLAST = 100 % with M. genavense (HM022216)	Nov 18 2010	1
10 8305	bison	Ridom = M. pulveris 100%	BLAST = 99% M. elephantis (AY859702, HM229788)	Sept 16 2010	0
10 8335	cow	Ridom 16S = M. chitae 99%	BLAST = 99% with M. confluentis (EU109298), M. chitae (EU109297)	August 26 2010	1
10 8384	goat	Ridom = MAC 100%	MEGA align 100% with M paratuberculosis (EF521906)	Oct 7 2010	1
10 8415	cow	Ridom = M. nonchromogenicum 100%	BLAST = 91% with M. scrofulaceum (GQ153305)	Oct 7 2010	0
10 8419 yellow	bison	Ridom 16S = M. pulveris (3 mismatches) 99%	BLAST = 99% M. elephantis (AY859702, HM229788)	"	0
10 8419 white	bison	Ridom 16S = M. gadium (96%)	BLAST = M. holsaticum (97% with AY859705)	Sept. 10 2010	0
10 8419 (rep 3)	bison	Ridom 16S = M. pulveris (3 mismatches) 99%	BLAST = 98% M. elephantis (AY859702, HM229788)	Sept. 24 2010	0
10 8435	turtle	Ridom 16S = M. intracellulare 100%	BLAST = 96% with M. indicus pranii (DQ437721), 96% with M. chimaera (EU770576), 96% with M. intracellulare (GQ153307)	Sept 30 2010	1
10 8532	deer	Ridom = MAC 100%	MEGA align = 100% with M paratuberculosis (EF521906)	Oct 21 2010	1
10 8545	ferret	16S rRNA seq unsuccessful, ITS seq = M. celatum 100%	BLAST = M. celatum (97% with JF346871, re-BLASTED on Sept 13 2011)	Sept. 10 2010	1
10 8669	elk	Ridom = M. terrae 100%	BLAST = 91% with M. malmoense (GQ153314)	Oct 21 2010	0
10 8681	bison	Ridom = M. pulveris 99%	BLAST = 99% with M. elephantis (AY859702)	Oct 7 2010	0

10 8723	cow	Ridom = M. pulveris 99%	BLAST = M. elephantis (99% with HM229788)	Sept 30 2010	0
10 8734	mandrill	Ridom = MAC 100%	BLAST / MEGA align = M hominissuis (100% with EF521911)	Nov 4 2010	1
10 8910	cow	Ridom = M. acapulcensis 99%	BLAST = M. flavescens (99% with AY859698)	Oct 14 2010	0
10 9098	cow	Ridom = M. fortuitum 100%	BLAST = M. senegalense (99% with AY262738), M. fortuitum (98% with AY147172)	Oct 14 2010	1
10 9199	red deer	Ridom = M. simiae 100%	BLAST = 96 % with M. parascrofulaceum (HM229796)	Nov 4 2010	0
10 9249	cow	Ridom = M. gadium 99%	BLAST = 97% with M. holsaticum (AY859705)	Nov 12 2010	0
10 9274	seahorse	Ridom = M. fortuitum (1 mismatch) 99%	BLAST = 96% with multiple species of Mycobacterium: M. fortuitum, M. farcinogenes, M. porcinum, etc	Sept 24 2010	1
10 9359 cfu	feral pig	Ridom = M. fortuitum complex 99%	BLAST = 99% with M. fortuitum (FJ418056), M. farcinogenes (AY262742), 98% with M. senegalense (AY262738)	Oct 21 2010	1
10 9449	cow	Ridom = M. intracellulare 100%	BLAST = 99% with M. indicus pranii (DQ437721), 99% with M. chimaera (EU770576), 99% with M. intracellulare (GQ153307)	Nov 4 2010	1
10 9484	llama	Ridom = M. intracellulare 100%	BLAST = 99% with M. indicus pranii (DQ437721), 99% with M. chimaera (EU770576), 99% with M. intracellulare (GQ153307)	Oct 21 2010	1
10 9526 1,2,3,4	dog	Ridom = M. abcessus / M. chelonae (x 4) 99%	BLAST = 100% with M. abcessus (x 4) (AY147164)	Nov 4 2010	1
11 0045	cow	Ridom = MAC 100%	BLAST / MEGA align = M hominissuis (100% with EF521911)	Nov 18 2010	1
11 0046	cow	Ridom = MAC 100%	BLAST / MEGA align = M hominissuis (100% with EF521911)	Nov 4 2010	1
11 0084	white-tailed deer	Ridom = M. abcessus / M. chelonae 99%	BLAST = 99% with M. abcessus (AY147164)	Nov 12 2010	1
11 0327	cow	Ridom = M. porcinum / M. fortuitum 99%	BLAST = M. porcinum (100% with AY262737)	Nov 26 2010	1
11 0470	cow	Ridom = M. porcinum / M. fortuitum 99%	BLAST = M. porcinum (100% with AY262737)	Dec 2 2010	1
11 1159	dog	Ridom = MAC 100%	BLAST / MEGA align = M hominissuis (100% with EF521911)	Nov 26 2010	1
10 9506	cat	Ridom = M. smegmatis 100%	BLAST = 100% with M. smegmatis (AY262735)	Dec 9 2010	1
11-00534	elephant	Ridom = MAC 100%	BLAST / MEGA align = M hominissuis (100% with EF521911)	Dec 16 2010	1

11-01094	cow	Ridom = M. fortuitum 100%	BLAST = M. fortuitum (99% with AY147173)	Dec 16 2010	1
11-01120	cow	Ridom = M. acapulcensis 100%	BLAST = M. flavescens (98% with AY859698)	Dec 16 2010	0
11-01449	cow	Ridom = M. fortuitum 100%	BLAST = M. fortuitum (100% with AY147173)	Dec 16 2010	1
11-01452	macaque	Ridom = M. septicum / M. peregrinum 100%	BLAST = M. peregrinum (99% with AY147166), M. alvei (AY859697)	Dec 16 2010	1
11-00079	cat	Ridom = M. intracellulare 100%	BLAST = M. marseillense (100% with EF584434)	Dec 16 2010	0
11-00854	cow	Ridom = M. septicum / M. peregrinum 100%	BLAST = M. peregrinum (98% with AY147166)	Dec 22 2010	1
11-01119	cow	Ridom = M. acapulcensis 100%	BLAST = M. flavescens (98% with AY859698)	Dec 22 2010	0
11-01454	macaque	Ridom = M. septicum / M. peregrinum 100%	BLAST = M. alvei (99% with AY859697)	Dec 22 2010	0
11-01603	cow	Ridom = M. abcessus, M. chelonae 99%	BLAST = several species at 97%, M. immunogenum (AY262739), M. massiliense (AY593981), M. chelonae (AY147163)	Dec 22 2010	0
11-00480	cow	Ridom = M. fortuitum 100%	BLAST = M. fortuitum (99% with AY147173)	Dec 29 2010	1
11-01436	cow	Ridom = M. smegmatis 100%	BLAST = M. smegmatis (99% with AY262735)	Dec 29 2010	1
11-00854-2	cow	Ridom = M. septicum / M. peregrinum 100%	BLAST = M. peregrinum (98% with AY147166)	Dec 29 2010	1
11-01528	elephant	Ridom = M. farcinogenes, fortuitum, senegalense 99%	BLAST = M. farcinogenes (99% with AY262742)	Dec 29 2010	1
11-01730	cow	Ridom = M. fortuitum, M. porcinum (100%)	BLAST = M. porcinum (100% with AY262737)	Dec 29 2010	1
11-01119 (2)	cow	Ridom = M. monacense 100%	BLAST = Mycobacterium sp. JLS (96% with CP000580), Mycobacterium sp. KMS (96% with CP000518), M. monacense (96% with HM229793)	Jan 6 2011	0
11-01428	fallow deer	Ridom = M. szulgai 99%	BLAST = M. interjectum (95% with HM022207)	Jan 6 2011	0
11-01519	cow	Ridom = M. kansasii 100%	BLAST = M. kansasii (97% with EU370227)	Jan 6 2011	1
11-01689	cow	Ridom = M. terrae 100%	BLAST = M. terrae (93% with JF346876; BLASTED Sept 13 2011 JH))	Jan 6 2011	1
11-00847	elk	Ridom = M. asiaticum 100%	BLAST = M. arosiense (93% with EU370532)	Jan 6 2011	0
11-00908-1	bison	Ridom = M. szulgai / conspicuum / intracellulare 98%	BLAST = M. montefiorense (97% with HM022209)	Jan 12 2011	0
11-01362	deer	Ridom = M. fortuitum complex 100%	BLAST = M. fortuitum (100% with AY147173)	Jan 12 2011	1

11-01364	deer	Ridom = M. abcessus / M. chelonae 100%	BLAST = M. bolletii (99% with AY859692)	Jan 12 2011	0
11-01368	deer	Ridom = M. abcessus / M. chelonae 100%	BLAST = M. bolletii (99% with AY859692)	Jan 12 2011	0
11-01375	deer	Ridom = M. fortuitum complex 100%	BLAST = M. fortuitum (100% with AY147173)	Jan 12 2011	1
11-01377	deer	Ridom = M. nonchromogenicum 100%	BLAST = M. wolinskyi (90% with AY262743)	Jan 12 2011	0
11-01400	cow	Ridom = M. farcinogenes / fortuitum / senegalense 100%	BLAST = M. farcinogenes (99% with AY262742) / M. senegalense (99% with AY262738) / M. fortuitum (99% with AY147173)	Jan 12 2011	1
11-01429	fallow deer	Ridom = M. septicum / M. peregrinum 100%	BLAST = M. peregrinum (99% with AY147166), M. alvei (AY859697)	Jan 12 2011	1
11-01456	monkey	Ridom = M. septicum / M. peregrinum 100%	BLAST = M. peregrinum (99% with AY147166), M. alvei (AY859697)	Jan 12 2011	1
11-00908-2	bison	Ridom = M. szulgai / conspicuum / intracellulare 98%	BLAST = M. montefiorense (97% with HM022209)	Jan 21 2011	0
11-01076	cow	Ridom = M. paraffinicum 99%	BLAST = M. nebraskense (97 % with GQ153310)	Jan 21 2011	0
11-01473	whitetail deer	Ridom = M. fortuitum 100%	BLAST = M. fortuitum (100% with AY147165)	Jan 21 2011	1
11-01527	elephant	Ridom = M. monacense 100%	BLAST = Mycobacterium sp. JLS (99% with CP000580), Mycobacterium sp. KMS (99% with CP000518), Mycobacterium sp. MCS (99% with CP000384), M. monacense (98% with HM229793)	Jan 21 2011	1
11-01952	cow	Ridom = M. smegmatis 100%	BLAST = M. smegmatis (100% with AY262735)	Jan 21 2011	1
11-02122	whitetail deer	Ridom = M. abscessus / M. chelonae 100%	BLAST = M. abscessus (99% with AY147164)	Jan 21 2011	1
11-02271	cow	Ridom = M. fortuitum 100%	BLAST = M. fortuitum (100% with AY147173)	Jan 21 2011	1
11-01359	deer	Ridom = M. intracellulare 99%	BLAST = M. colombiense (97% with GQ153308)	Jan 27 2011	0
11-01376	deer	Ridom = M. intracellulare 100%	BLAST = M. chimera (99% with EU770576)	Jan 27 2011	0

11-01690	cow	Ridom = M. fortuitum 100%	BLAST = M. fortuitum (99% with AY147173)	Jan 27 2011	1
11-01740	whitetail deer	Ridom = M. fortuitum 100%	BLAST = M. fortuitum (99% with AY147173)	Jan 27 2011	1
11-01846 - 2	whitetail deer	Ridom = MAC 100%	BLAST / MEGA align = M hominissuis (100% with EF521911)	Jan 27 2011	1
11-01852	whitetail deer	Ridom = M. gastri / M. kansasii 100%	BLAST = M. kansasii (100 % with our M. kansasii ATCC 12478)	Jan 27 2011	1
11-01864	whitetail deer	Ridom = MAC 100%	BLAST / MEGA align = M hominissuis (100% with EF521911)	Jan 27 2011	1
11-01903	cow	Ridom = MAC 100%	BLAST / MEGA align = M hominissuis (100% with EF521911)	Jan 27 2011	1
11-01905	cow	Ridom = MAC 100%	BLAST / MEGA align = M hominissuis (100% with EF521911)	Jan 27 2011	1
11-01912	cow	Ridom = M. paraffinicum 99%	BLAST = M. nebraskense (98 % with GQ153310)	Jan 27 2011	0
11-01786	sheep	Ridom = MAC 100%	MEGA align = M. paratuberculosis (100% with EF521906)	Febr 4 2011	1
11-01904	cow	Ridom = MAC 100%	BLAST / MEGA align = M hominissuis (100% with EF521911)	Febr 4 2011	1
11-02429	pig	Ridom = MAC 100%	BLAST / MEGA align = M hominissuis (100% with EF521911)	Febr 4 2011	1
11-00959	turtle	Ridom = M. terrae 99.5%	BLAST = M. terrae 94%	Febr 10 2011	1
11-00995	whitetail deer	Ridom = MAC 100%	MEGA align = M. hominissuis / M avium / M paratuberculosis (99% with EF521911. EF521907, & EF521906)	Febr 10 2011	1
11-01876	whitetail deer	Ridom = M. fortuitum 100%	BLAST = M. fortuitum (99% with AY147173)	Febr 10 2011	1
11-01893	whitetail deer	Ridom = M. asiaticum 99.2%	BLAST = M. montefiorense (94% with HM022209)	Febr 10 2011	0
11-02407	elk	Ridom = M. abcessus / M. chelonae 99.7%	BLAST = M. abcessus (99% with AY147164)	Febr 10 2011	1
11-00880	pig	Ridom = MAC 100%	MEGA = M. paratuberculosis (100% with EF521906)	Febr 16 2011	1
11-00986	deer	Ridom = MAC 100%	MEGA = 99% with MAP, MAH, MAA (EF521906, EF521907, EF521911)	Febr 16 2011	1
11-00989	deer	Ridom = M. intracellulare 100%	BLAST = M. chimaera (99% with EU770576) & M. intracellulare (99% with HM807413)	Febr 16 2011	1

11-01010	deer	Ridom = MAC 100%	BLAST / MEGA align = M hominissuis (100% with EF521911)	Febr 16 2011	1
11-01019	deer	Ridom = MAC 100%	MEGA = 99% with MAP, MAH, MAA (EF521906, EF521907, EF521911)	Febr 16 2011	1
11-01031	deer	Ridom = MAC 100%	MEGA = 99% with MAP, MAH, MAA (EF521906, EF521907, EF521911)	Febr 16 2011	1
11-01354	cow	Ridom = M. simiae 99%	BLAST = M. montefiorense (97% with HM022209)	Febr 16 2011	0
11-01851	deer	Ridom = M. septicum / M. peregrinum 100%	BLAST = M. septicum (99% with AY772165)	Febr 16 2011	1
11-02019	elephant	Ridom = M. intracellulare 99%	BLAST = M. colombiense (98% with GQ153308)	Febr 16 2011	0
11-02023	cow	Ridom = MAC 100%	MEGA = 99% with MAP, MAH, MAA (EF521906, EF521907, EF521911)	Febr 16 2011	1
11-02419	elk	Ridom = M. simiae 99%	BLAST = M. montefiorense (97% with HM022209)	Febr 16 2011	0
11-02504	cow	Ridom = M. nonchromogenicum 100%	BLAST = M. wolinskyi (91% with AY262743)	Febr 16 2011	0
11-01048	deer	Ridom = M. intracellulare 100%	BLAST = M. intracellulare (100 % with GQ153307, 99% M. chimaera (99% with EU770576)	Febr 25 2011	1
11-02260	cow	Ridom = M. porcinum / M. fortuitum 100%	BLAST = M. porcinum (100% with AY262737)	Febr 25 2011	1
11-02265	cow	Ridom = M. goodii 99%	BLAST = M. smegmatis (98% with AJ605718)	Febr 25 2011	0
11-01007	deer	Ridom = M. gastri / M. kansasii 100%	BLAST = M. kansasii (100% with HQ880687)	March 3 2011	1
11-01799	bison	Ridom = M. neoaurum 100%	BLAST = M. neoaurum (99 % with FJ172335, only 638 nt)	March 3 2011	1
11-01888 buff	deer	Ridom = MAC 100%	MEGA = 99% with MAP, MAH, MAA (EF521906, EF521907, EF521911)	March 3 2011	1
11-01888 yellow	deer	Ridom = M. gastri / M. kansasii 100%	BLAST = M. kansasii (100% with HQ880687)	March 3 2011	1
11-02218	deer	Ridom = M. terrae 99%	BLAST = M. terrae (96% with EU591502, 632 nt)	March 3 2011	1
11-02433	cow	Ridom = M. septicum / peregrinum 100%	BLAST = M. peregrinum (100 % with AY147166)	March 3 2011	1
11-02685	elephant	Ridom = M. gadium 96 %	BLAST = M. holsaticum (98% with AY859705)	March 3 2011	0
11-02744	cow	Ridom = M. pulveris 99%	BLAST = M. elephantis (99% with HM229788)	March 3 2011	0

11-02804	deer	Ridom = MAC 100%	BLAST / MEGA align = M hominissuis (100% with EF521911)	March 3 2011	1
11-02829	deer	Ridom = MAC 100%	MEGA = 99% with MAP, MAH, MAA (EF521906, EF521907, EF521911)	March 3 2011	1
11-02433	cow	Ridom = M. septicum / peregrinum 100%	BLAST = M. peregrinum (100 % with AY147166)	March 3 2011	1
11-03055	cow	Ridom = M. septicum / peregrinum 100%	BLAST = M. peregrinum (100 % with AY147166)	March 3 2011	1
11-03450	frog	Ridom = M. marinum / M. ulcerans 100%	BLAST = M. marinum / M. ulcerans (99% with CP000854 / CP000325)	March 3 2011	1
11-03451	seal	Ridom = M. fortuitum 100%	BLAST = M. fortuitum (100% with AY147173)	March 3 2011	1
11-03521	dog	Ridom = MAC 100%	BLAST / MEGA align = M hominissuis (100% with EF521911)	March 3 2011	1
11-01025	deer	Ridom = M. gastri / M. kansasii 100%	BLAST = M. kansasii (100% with HQ880687)	March 10 2011	1
11-02604	bison	Ridom = M. simiae 100%	BLAST = M. saskatchewanense / M. montefiorense (97% with HM022212 and HM022209)	March10 2011	0
11-02849	deer	Ridom = MAC 100%	BLAST / MEGA align = M hominissuis (100% with EF521911)	March10 2011	1
11-02859	deer	Ridom = MAC 100%	BLAST / MEGA align = M hominissuis (100% with EF521911)	March 10 2011	1
11-02879	deer	Ridom = MAC 100%	BLAST / MEGA align = M hominissuis (100% with EF521911)	March10 2011	1
11-030346	elephant	Ridom = M. septicum / M. peregrinum 100%	BLAST = M. septicum (99% with AY772165)	March 10 2011	1
11-02382	cow	Ridom = M. nonchromogenicum 100%	BLAST = M. wolinskyi (91% with AY262743)	March 17 2011	0
11-02950	cow	Ridom = M. thermoresistans 100%	BLAST = M. wolinskyi (91% with AY262743)	March 17 2011	0
11-03332	cow	Ridom = M. fortuitum complex 100%	BLAST = M. fortuitum (99% with AY147173)	March 17 2011	1
11-04744	cat	Ridom = M. fortuitum complex 100%	BLAST = M. fortuitum (99% with AY147173)	March 17 2011	1
11-03469B	monkey	Ridom = M. gastri / M. kansasii 100%	BLAST = M. kansasii (100% with HQ880687, 640 of 640 bp)	March 31 2011	1
11-1013	deer	Ridom = M. gastri / M. kansasii 100%	BLAST = M. kansasii (100% with HQ880687)	March 24 2011	1

11-1049	deer	Ridom = MAC 100%	BLAST / MEGA align = M hominissuis (100% with EF521911)	March 24 2011	1
11-2872	deer	Ridom = M. alvei 100%	BLAST = M. setense (100% with HM807426)	March 24 2011	0
11-2909	deer	Ridom = MAC 100%	BLAST / MEGA align = M hominissuis (100% with EF521911)	March 24 2011	1
11-2924	cow	Ridom = MAC 100%	BLAST / MEGA align = M hominissuis (100% with EF521911)	March 24 2011	1
11-4616	cat	Ridom = MAC 100%	BLAST / MEGA align = M hominissuis (100% with EF521911)	March 24 2011	1
11-01049	deer	Ridom = MAC 100%	BLAST / MEGA align = M hominissuis (100% with EF521911)	March 31 2011	1
11-3005	elk	Ridom = M. intermedium 99.7 %	BLAST = M. intermedium (100% with HM 022208)	March 31 2011	1
11-4248	cat	Ridom = M. thermoresistable 100%	BLAST = M. wolinskyi (91% with AY262743)	March 31 2011	0
11-02750	elephant	Ridom = M. paraffinicum 100%	BLAST = M. paraffinicum (96% with GQ153304)	April 7 2011	1
11-02759	elephant	Ridom = M. terrae 99%	BLAST = M. terrae (93% with JF346876)	April 7 2011	1
11-02995	cow	Ridom = M. fortuitum complex 100%	BLAST = M. fortuitum (100% with AY147173)	April 7 2011	1
11-03148	opossum	Ridom = MAC 100%	BLAST / MEGA align = M hominissuis (100% with EF521911)	April 7 2011	1
11-03813	rhesus monkey	Ridom = M. gastri / M. kansasii 100%	BLAST = M. kansasii (100% with with HQ880687)	April 7 2011	1
11-03276	elk	Ridom = M. septicum / M. peregrinum 100%	BLAST = M. septicum (99% with AY772165)	April 14 2011	1
11-03453, 11-03454, 11-03455	tapir, Malayan	Ridom = M. triviale 97%, M. gordonae 100%	3453 & 3454: BLAST = Myco sp. NLA001000736 (100% with HM627012); 3455: M. gordonae 100% (691 nt with 694 of EU597586)	April 14 2011, April 21 2011	1
11-04216	cow	Ridom = M. smegmatis 100%	BLAST = M. smegmatis (100% with AY262735)	April 14 2011	1
11-04803	toad, fire belly	Ridom = M. immunogenum 99%	BLAST = M. immunogenum (99% with AY262739)	April 14 2011	1
11-02650	deer, fallow	Ridom = M. simiae 100%	BLAST = M. parascrofulaceum (96% with HM229796)	April 21 2011	0
11-02817	deer	Ridom = M. alvei 100%	BLAST = M. setense (100% with HM807426)	April 21 2011	0

11-02880	deer	Ridom = MAC 100%	BLAST / MEGA align = M hominissuis (100% with EF521911)	April 21 2011	1
11-05038	dog	Ridom = MAC 100%	BLAST / MEGA align = M hominissuis (100% with EF521911)	April 21 2011	1
11-05219	dog	Ridom = M. abcessus / M/ chelonae 100%	BLAST = M. bolletii (99% with AY859692)	April 21 2011	0
11-05296	cat	Ridom = MAC 100%	BLAST / MEGA align = M hominissuis (100% with EF521911)	April 21 2011	1
11-03160	opossum	Ridom = MAC 100%	BLAST / MEGA align = M hominissuis (100% with EF521911)	April 28 2011	1
11-05220	cat	Ridom = M. goodii 100 %	BLAST = M. goodii (99% with AY262736)	May 5 2011	1
11-02880	deer	Ridom = MAC 100%	BLAST / MEGA align = M hominissuis (100% with EF521911)	May 13 2011	1
11-05104	cow	Ridom = M. pulveris 99%	BLAST = M. elephantis (98% with AY859702)	May 13 2011	0
11-05113	cow	Ridom = M. monacense 97%	BLAST = Myco spp. JLS, KMS, MCS all at 95%, M. monacense at 94% (HM229793)	May 26 2011	1
11-05598	cow	Ridom = M. nonchromogenicum 100%	BLAST = M. scrofulaceum at 90% (GQ153305)	May 26 2011	0
11-05929	dog	Ridom = MAC 100%	BLAST / MEGA align = M hominissuis (100% with EF521911)	May 26 2011	1
11-05858	cow	Ridom = MAC 100%	BLAST = M. avium (100% with EF521907)	May 26 2011	1
11-05160	cow	Ridom = M. fortuitum 100%	BLAST = M. fortuitum (100% with AY147173)	May 26 2011	1
11-05565	elk	Ridom = M. intracellulare 100%	BLAST = M. colombiense (97% with GQ153308)	May 26 2011	0
11-05100	cow	Ridom = M. lentiflavum 99%	BLAST = M. palustre (HM022210)	June 2 2011	0
11-05105	cow	Ridom = M. triplex 96%	BLAST = M. triplex (96% with HM022214)	June 2 2011	1

* the RIDOM 16S rRNA database only permits assignation to the MAC, not to species / subspecies level for most members of this complex
