# Supplementary material for: Identification of Mycobacterium spp. of veterinary importance using rpoB gene sequencing
Source: BMC Vet Res. 2011 Nov 25;7:77. doi: 10.1186/1746-6148-7-77 (PMC3251535; doi:10.1186/1746-6148-7-77)

Additional File 2

ABI 3500XL electropherogram depicting the base calls for the region of the *rpoB* gene of *M. avium* subsp. *silvaticum* ATCC 49884 where nucleotide 2,541 (using the *M. avium* subspecies *paratuberculosis* rpoB K10 strain numbering convention) presents as a cytosine (outlined in the yellow box).

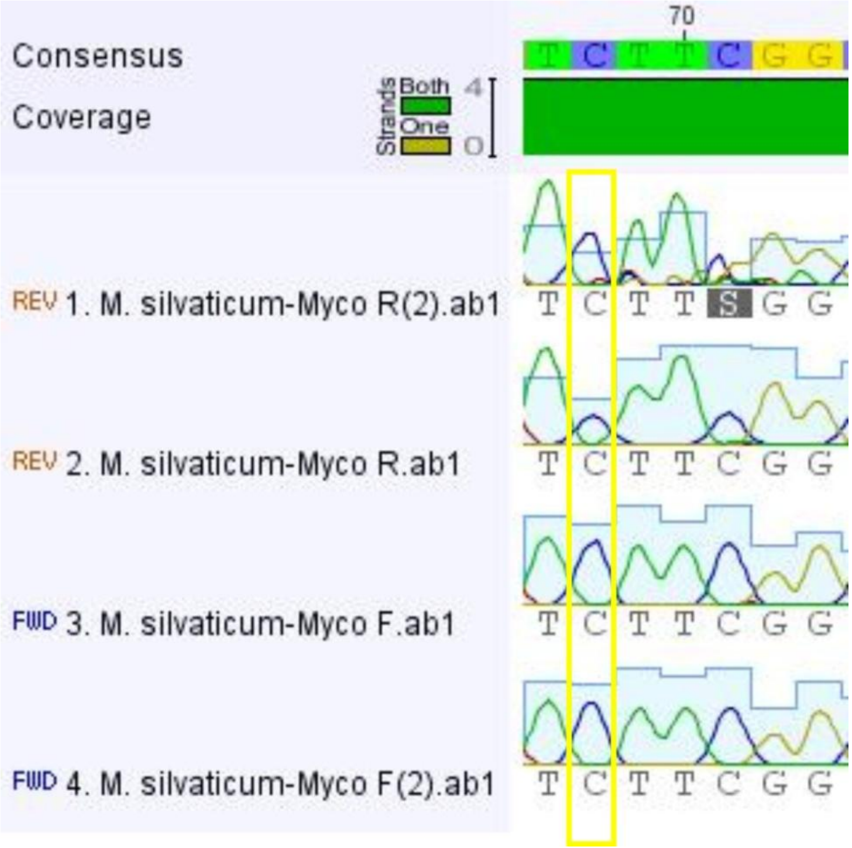

Supplement: Additional file 2 — Presence of a cytosine residue in the rpoB sequence of M. avium subsp. silvaticum ATCC 49884. A pdf file of a ABI 3500XL electropherogram depicting the base calls for the region of the rpoB gene of M. avium subsp. silvaticum ATCC 49884 where nucleotide 2, 541 (using the M. avium subspecies paratuberculosis rpoB K10 strain numbering convention) presents as a cytosine (outlined in the yellow box). [file 1746-6148-7-77-S2.PDF]
